# 2024 Royal Australian College of General Practitioners and Healthy Bones Australia guideline for osteoporosis management and fracture prevention in postmenopausal women and men over 50 years of age

**DOI:** 10.5694/mja2.52637

**Published:** 2025-03-25

**Authors:** Peter Wong, Weiwen Chen, Dan Ewald, Christian Girgis, Morton Rawlin, John Tsingos, Justine Waters

**Affiliations:** ^1^ Westmead Hospital Sydney NSW; ^2^ University of Sydney Sydney, NSW; ^3^ Garvan Institute Sydney NSW; ^4^ St Vincent's Hospital Sydney NSW; ^5^ University Centre for Rural Health University of Sydney Lismore NSW; ^6^ Lennox Head Medical Centre Lennox Head NSW; ^7^ Macedon Medical Centre Melbourne VIC; ^8^ Souths Juniors Medical Centre Sydney NSW; ^9^ University of Technology Sydney Sydney NSW

**Keywords:** Osteoporosis, Fractures, bone, Guidelines as topic, General practice

## Abstract

**Introduction:**

This updated guideline replaces the previous Royal Australian College of General Practitioners and Osteoporosis Australia (now, Healthy Bones Australia) guideline from 2017. The accumulation of high quality evidence supporting improvements in clinical practice over the past five years, need for expert consensus and opinion, and new developments in pharmacological management of osteoporosis, especially the role of osteoanabolic therapies, prompted this update. The aim was to provide clear, evidence‐based recommendations to assist Australian general practitioners in managing patients over 50 years of age with poor bone health. However, it is useful for any health care professional caring for people with poor bone health and for health administrators and bureaucrats responsible for resource provision and allocation.

**Main recommendations:**

- Earlier recognition of poor bone health using clinical risk factors, and use of an absolute fracture risk assessment tool, particularly FRAX (https://fraxplus.org/), is encouraged.
- Widespread population‐based osteoporosis screening is not recommended in Australia due to lack of supporting evidence.
- It is important to recognise patients with “imminent” or “very high” fracture risk, as this is a group in whom to consider early osteoanabolic therapy.
- Calcium and vitamin D supplementation are more effective in reducing fracture risk when given to individuals who have calcium and vitamin D deficiency (not to healthy non‐institutionalised individuals).

**Changes in assessment and management as a result of the guideline:**

This guideline provides recommendations for the use of fracture risk assessment tools, particularly FRAX, for risk stratification, addresses the risk of rebound vertebral fracture following denosumab cessation, discusses removal of strontium as a therapy, clarifies “imminent” or “very high” fracture risk in patients and highlights the importance of calcium and vitamin D status, and the early use of osteoanabolic therapies. The full guideline is freely available at https://www.racgp.org.au/clinical‐resources/clinical‐guidelines/key‐racgp‐guidelines/view‐all‐racgp‐guidelines/osteoporosis/executive‐summary.

Osteoporosis is characterised by low bone mineral density (BMD) and micro‐architectural deterioration of bone tissue, leading to decreased bone strength and increased fragility and fracture risk. Osteoporotic (fragility) fractures usually follow falls from a standing height, or less, in individuals with decreased bone strength, and involve low or minimal trauma. Bone mineral density can be reliably measured by scanning the skeleton using dual‐energy x‐ray absorptiometry (DXA). Deterioration of skeletal tissue proceeds with no symptoms until a symptomatic fracture occurs. The condition is therefore under‐recognised and affected individuals are undertreated.^1^, ^2^

Fracture‐related morbidity can arise from pain, reduced mobility, loss of function, and consequent reduced quality of life.^3^ Many patients are unable to live independently following a hip fracture. Long term morbidity is associated with symptomatic osteoporotic fractures at almost all anatomic sites.^3^ Mortality in the first year after a major minimal trauma fracture in people aged over 60 years is up to threefold higher than in age‐matched non‐fracture populations for people with hip fracture and up to twofold higher for other major fracture types^4^, ^5^ — major fractures include those of the pelvis, hip, distal forearm, humerus, and vertebrae. Prompt diagnosis and optimal treatment of osteoporosis prevents further fractures and reduces mortality.^6^, ^7^, ^8^

The Australian Government Department of Health and Aged Care contracted Healthy Bones Australia (formerly Osteoporosis Australia), which is a national not‐for‐profit organisation and a leading national bone health consumer body, to update the previous Royal Australian College of General Practitioners (RACGP) and Osteoporosis Australia (now, Healthy Bones Australia) 2017 guideline for osteoporosis management.^9^ The accumulation of high quality evidence supporting improvements in clinical practice over the past five years, the need for expert consensus and opinion, and new developments in pharmacological management of osteoporosis, especially the role of osteoanabolic therapies such as romosozumab and teriparatide, prompted this update.

The updated guideline^10^ was designed to provide clear, evidence‐based recommendations to assist Australian general practitioners in managing patients over 50 years of age with poor bone health (osteopenia and osteoporosis). Its purpose was to support, not replace, clinical decision making in the individual patient, and to assist busy general practitioners in achieving better patient outcomes by:

- preventing the first fracture;
- diagnosing osteoporosis early to allow prompt bone health management;
- identifying undiagnosed patients following a first fracture to prevent subsequent fractures; and
- managing secondary causes of poor bone health.

## Methods

Recommendations in the previous (second) edition^9^ were based on critical analysis of published, peer‐reviewed evidence from 2006 to 2016, following a systematic review of available evidence. Every section in the updated guideline^10^ was reviewed and updated by a Subject Matter Adviser (Supporting Information, table 1) with subspecialty expertise in that topic using new peer‐reviewed evidence published from 2017. Focused literature searches were undertaken in subject areas that the Guideline Review Committee (PW, WC, DE, CG, MR, JT, JW) felt needed particular attention. These included fracture risk assessment tools, the frequency of DXA monitoring, patients at “imminent” or “very high” fracture risk, and pharmacological therapies. For these areas, the Guideline Review Committee provided specific keywords to the RACGP team, who searched the following databases: PubMed, Medline, National Institute for Heath and Care Excellence (NICE), Cochrane Database of Systematic Reviews and Cochrane Central Register of Controlled Trials (CENTRAL), Scottish Intercollegiate Guidelines Network (SIGN), Trip Database, and Google. Filters were applied in Ovid Medline to identify randomised controlled trials, systematic reviews and meta‐analyses. Other filters applied included men and women older than 45 years of age and studies reporting outcomes of fracture and/or BMD. As far as possible, evidence to support recommendations covering pharmacological and other therapeutic interventions was restricted to studies with fracture as a primary outcome. However, for some interventions, evidence meeting this criterion was sparse, or of variable quality, and high quality studies with BMD as a primary outcome were used if, in the opinion of the Guideline Review Committee, the data could be used to support recommendations.

Each of the 45 recommendations was given a final grading from A to D according to the National Health and Medical Research Council grades of recommendation.^11^ The grading represents the overall strength of evidence and reflects the confidence with which clinicians can apply a recommendation in a clinical situation. However, they should be used in conjunction with clinical judgement and individual patient context and preferences. The recommendations do not cover complex medical conditions with comorbidities, nor are they a substitute for individualised specialist advice and/or consultation, which may be required for optimal patient care.

Where insufficient evidence was available, or where the quality of evidence did not meet minimum requirements, recommendations were developed through the Guideline Review Committee's unanimous consensus, cognisant of the complexities and time constraints of a busy general practitioner.

## Conflicts of interest

All members of the Guideline Review Committee were asked to declare any potential conflicts of interest. This was updated at each meeting and is available as a supplementary document.^10^ The management of the conflicts of interest was undertaken as per Guideline International Network (GIN)^12^ principles and are explained in the supplementary document.^10^

### Consultation and endorsement by the RACGP

Due to resource and time restrictions, consultation was focused on Healthy Bones Australia, stakeholders and review by the intended guideline users, namely general practitioners. The Guideline Review Committee was particularly aware of the importance of clear and pragmatic advice for busy general practitioners in everyday clinical practice. This guide was reviewed by general practice subject matter experts (DE, MR, JT) on the Guideline Review Committee and the RACGP's Expert Committee – Quality Care and endorsed by the RACGP Board.

## Recommendations

The guideline recommendations are outlined in Box 1. The full guideline is freely available at https://www.racgp.org.au/clinical‐resources/clinical‐guidelines/key‐racgp‐guidelines/view‐all‐racgp‐guidelines/osteoporosis/executive‐summary. A high resolution summary flowchart is available at https://www.racgp.org.au/getattachment/f31c6529‐96f0‐4840‐8f41‐c98bd5e4fad7/attachment.aspx?disposition=inline (Box 2). Significant updates since the 2017 guideline are discussed next.

Box 1 **Recommendations^10^**

**Table:** 

Section	No.	Recommendation	Grade*
**1. Risk factors, fracture risk assessment and case finding**			
1.1 Identifying patients to investigate for osteoporosis	1	All individuals over the age of 50 years who sustain a fracture following minimal trauma (such as a fall from standing height, or less) should be considered to have a presumptive diagnosis of osteoporosis.	A
	2^†^	Conduct a clinical risk factor assessment in postmenopausal women and men over the age of 50 years with one or more major risk factors for minimal trauma fracture to guide bone mineral density (BMD) measurement and prompt timely referral and/or drug treatment.	A
	3	A presumptive diagnosis of osteoporosis can be made in a patient with a vertebral fracture or hip fracture in whom there is no history of significant trauma. Caution regarding diagnosis and treatment should be exercised if only a single mild vertebral deformity (height loss) is detected, especially in a patient under the age of 60 years.	B
1.2 Measurement of bone mineral density	4^†^	Measure BMD by dual‐energy x‐ray absorptiometry (DXA) scanning on at least two skeletal sites, including the lumbar spine and hip, unless these sites are unsuitable (eg, hip prosthesis).	A
1.3 Assessment of absolute fracture risk	5^†^	Assessment of absolute fracture risk, using the Fracture Risk Assessment Tool (FRAX; https://fraxplus.org/) may be useful in assessing the need for treatment in individuals who do not clearly fit the established criteria.	B
	6^†^	Patients with a very high and/or imminent fracture risk should be promptly referred to a specialist for consideration of osteoanabolic therapy as first line treatment.	C
1.4 Case finding	7	Those aged > 50 years with a current or prior minimal trauma fracture should be assessed and appropriately treated.	A
	8^†^	For those aged > 50 years with lifestyle and non‐modifiable risk factors (eg, parent with hip fracture), use FRAX to calculate absolute fracture risk. When FRAX risk for major osteoporotic fracture (MOF) is ≥ 10%, refer for DXA. If the risk of MOF is < 10%, DXA is not recommended. Re‐stratify risk with FRAX after DXA using BMD reading and treat when: ‣ BMD T‐score is ≤ ‐2.5; ‣ BMD T‐score is between ‐1.5 and ‐2.5 and the FRAX risk for MOF is ≥ 20% and/or the hip fracture risk is ≥ 3%.	D
	9^†^	For those aged > 50 years with diseases, chronic conditions and/or medications associated with increased fracture risk, refer for BMD assessment by DXA. Re‐stratify risk with FRAX after DXA using BMD reading and treat when: ‣ BMD T‐score is ≤ ‐2.5; ‣ BMD T‐score is between ‐1.5 and ‐2.5 and the FRAX risk for MOF is ≥ 20% and/or the hip fracture risk is ≥ 3%.	C
	10	There is insufficient evidence to recommend population‐based systematic screening with BMD measurement for reduction of osteoporotic fractures in Australia, and case finding is recommended.	B
**2. General bone health maintenance and fracture prevention**			
2.1 Calcium, protein and vitamin D	11^†^	**For generally healthy older people:** Although the absolute benefit of calcium and vitamin D supplements in short term (less than six years) studies for fracture reduction is low, there is good evidence that adequate calcium intake and vitamin D status are important for long term maintenance of bone and muscle function.	C
	12^†^	**For frail and institutionalised older people:** Calcium and vitamin D supplementation, together with adequate protein intake, are recommended for fracture prevention. Optimisation of calcium and vitamin D should be the standard of care for this group of people.	B
	13^†^	**For people taking osteoporosis treatments:** Calcium supplements should be recommended if their dietary calcium intake is less than 1300 mg per day. Vitamin D supplements should be recommended to correct low serum vitamin D levels (25‐hydroxyvitamin D < 50 nmol/L).	C
	14^†^	For most people with olive or pale brown skin, with no other risk factors and who are at intermediate risk of skin cancer, a few minutes of sunlight exposure towards the middle of the day, with time depending on latitude, season and skin area exposed, followed by further sun protection measures should maintain vitamin D levels. People with dark skin at low risk of skin cancer have less need for sun protection, but require more time outdoors to avoid vitamin D deficiency. People at high risk of skin cancer need sun protection most of the year, which may limit vitamin D synthesis. The use of sunscreen, in practice, does not greatly affect vitamin D status.	B
2.2 Reducing falls	15	Opportunistic case finding should be undertaken as per the recommended algorithm^1^ to identify older people at risk of falls and fall‐related injury.	A
	16	Offer further assessment and/or interventions to prevent falls based on the level of risk identified.	A
2.3 Exercise	17	**Exercises recommended to reduce fracture risk:** Muscle resistance (strength) training should be regular (at least twice a week), moderate to vigorous, and progressive. Weight‐bearing impact exercises should be performed most days (at least 50 moderate impacts) and include moderate to high loads in a variety of movements in different directions. Balance training activities should be challenging. Limit prolonged sitting (sedentary behaviour).	B
	18	Exercise programs for very frail older institutionalised people and those with a high vertebral fracture risk should be supervised, modified and tailored to minimise the potential to increase the risk of falls, injury and vertebral fractures.	C
	19	Prescribe extended and supervised exercise therapy, including targeted resistance and challenging balance training, after hip fracture to improve mobility, strength and physical performance and to reduce falls risk.	B
	20	Evidence for the benefits of exercise after vertebral and non‐hip fractures is limited, but suggests supervised resistance training will build bone once a fracture has healed to the same extent as in non‐fractured patients. For people with a vertebral fracture, exercises to strengthen back muscles, enhance flexibility and improve posture, as well as to reduce falls risk, should be considered.	D
**3. Pharmacological approaches to prevention and treatment**			
3.1 Bisphosphonates	21^†^	Bisphosphonate therapy (alendronate, risedronate or zoledronate) should be considered for the primary prevention of vertebral fractures in women with osteopenia who are at least ten years postmenopause.	B
	22^†^	Bisphosphonate therapy is recommended for reducing the risk of vertebral and non‐vertebral fractures in postmenopausal women and men over the age of 50 years at high risk of fracture (those with osteoporosis by BMD criteria, or prior minimal trauma fracture).	A (women), C (men)
	23^†^	Reconsider the need to continue bisphosphonate therapy after five to ten years in postmenopausal women and men over the age of 50 years with osteoporosis who have responded well to treatment (T‐score ≥ ‐2.5 and no recent fractures). If BMD remains low (T‐score ≤ ‐2.5) and/or there are incident fragility fractures, continue treatment. Treatment should be restarted if there is bone loss, especially at the hip, or if a further minimal trauma fracture is sustained.	D
3.2 Denosumab	24^†^	Denosumab is recommended for the treatment of osteoporosis in postmenopausal women at high risk of minimal trauma fracture.	A
	25^†^	Denosumab may be considered as an alternative to bisphosphonates for the treatment of men at increased risk of minimal trauma fracture.	B
	26^†^	Denosumab therapy should not be interrupted. If denosumab needs to be ceased, patients should be transitioned to bisphosphonate therapy for a minimum of 12 months.	C
3.3 Romosozumab	27^†^	Romosozumab is recommended as first line therapy for osteoporosis treatment in postmenopausal women at very high risk of minimal trauma fracture.	A
	28^†^	Romosozumab is recommended as first line therapy for osteoporosis treatment in men at very high risk of minimal trauma fracture.	C
3.4 Menopausal hormone therapy	29^†^	Consider oestrogen replacement therapy to reduce the risk of fragility fractures in postmenopausal women within ten years of menopause. The increased risk of adverse events associated with treatment should be carefully weighed against benefits.	A
	30^†^	Selective oestrogen receptor modulators (SERMs) should be considered as a treatment option for postmenopausal women with osteoporosis where vertebral fractures are the major osteoporosis risk (based on low spine BMD and/or an existing vertebral fracture) and where other agents are poorly tolerated. SERMs may be particularly useful in younger postmenopausal women at risk of vertebral fracture with a prior or family history of breast cancer.	A
3.5 Recombinant human parathyroid hormone	31	Recombinant human parathyroid hormone (teriparatide) treatment is recommended to reduce fracture risk in postmenopausal women with osteoporosis who have sustained a subsequent fracture while on antiresorptive therapy, or in those at very high fracture risk.	A
	32	Recombinant human parathyroid hormone (teriparatide) treatment is recommended to reduce fracture risk in men aged over 50 years with osteoporosis who have sustained a subsequent fracture while on antiresorptive therapy, or in those at very high fracture risk.	C
**4. Ongoing monitoring**			
4.1 Ongoing monitoring	33	Regularly reassess fracture risk and the requirement for anti‐osteoporotic therapy in patients not receiving therapy, but who remain at increased fracture risk.	C
	34	Clinically review all patients three to six months after initiating pharmacological therapy for osteoporosis, and every six to 12 months thereafter for medication side effects and therapy adherence.	C
	35	Measurement of bone turnover markers should be confined to specialist practice. Measurement of bone turnover markers may be useful for monitoring medication adherence and efficacy and for evaluation of secondary causes of bone loss.	D
**5. Special issues**			
5.1 Management of osteoporosis in frail and older people (> 75 years of age)	36	Consider a multifactorial approach (environment, pharmacological treatments, exercise, nutrition) to reduce falls and fracture risk.	C
5.2 Bone loss associated with aromatase inhibitor therapy for breast cancer and androgen deprivation therapy for prostate cancer	37	All women commencing aromatase inhibitor therapy should have baseline assessment of fracture risk before commencing therapy, including clinical risk factors, biochemistry and BMD (DXA) measurement, with ongoing monitoring based on risk factors.	A
	38	Women commencing aromatase inhibitor therapy who fall within one of the following two categories should commence antiresorptive therapy unless contraindicated: ‣ age ≥ 70 years with BMD T‐score ≤ ‐2.0; ‣ age > 50 years with a minimal trauma fracture (including radiological vertebral fracture) or a high estimated ten‐year fracture risk. There is limited evidence specific to women receiving aromatase inhibitors to guide firm recommendations outside these criteria, especially in pre‐menopausal women.	A
	39	The duration of anti‐resorptive treatment in women undergoing, or who have completed, aromatase inhibitor therapy should be individualised and based on absolute fracture risk.	D
	40	General measures to prevent bone loss should be implemented in all women commencing aromatase inhibitor therapy.	C
	41	All men commencing androgen deprivation therapy (ADT) should have a baseline assessment of fracture risk, including BMD assessment by DXA.	A
	42	All men receiving ADT with a history of minimal trauma fracture should be commenced on anti‐resorptive therapy, unless contraindicated.	A
	43	Bone health should be reviewed every one to two years in men on continuous ADT.	C
	44	General measures to prevent bone loss should be implemented in all men commencing ADT.	C
5.3 Medication‐related osteonecrosis of the jaw (MRONJ)	45^†^	MRONJ is a rare complication of osteoporosis therapy and most patients will not be at increased risk of MRONJ. Consider patient risk of MRONJ before starting osteoporosis therapy and ensure that high risk patients receive dental review before therapy initiation. Given the long in vivo half‐life of bisphosphonates, there is little benefit to their cessation before dental extraction. Invasive dental procedures in patients on denosumab should be performed just before the next six‐monthly injection because the in vivo effect on bone suppression will be waning.	C

* National Health and Medical Research Council grades of recommendations: A = body of evidence can be trusted to guide practice; B = body of evidence can be trusted to guide practice in most situations; C = body of evidence provides some support for recommendations, but care should be taken in its application; D = body of evidence is weak and recommendations must be applied with caution. † Recommendations underwent a focused and detailed search of the published literature during which multiple databases were interrogated to identify publications subsequent to the previous edition (ie, since 2016). These were then reviewed by a Subject Matter Adviser (Supporting Information, table 1) with subspecialty topic expertise and the relevant chapters updated. The final draft of the chapters underpinning relevant recommendations was then reviewed by the Guideline Review Committee (PW, WC, DE, CG, MR, JT, JW) and discussed at several face‐to‐face and online meetings. All other recommendations have been updated by at least one Subject Matter Adviser with subspecialty expertise in the area and reviewed by the Guideline Review Committee at two face‐to‐face meetings.

Box 2 **Osteoporosis risk assessment, diagnosis and management**

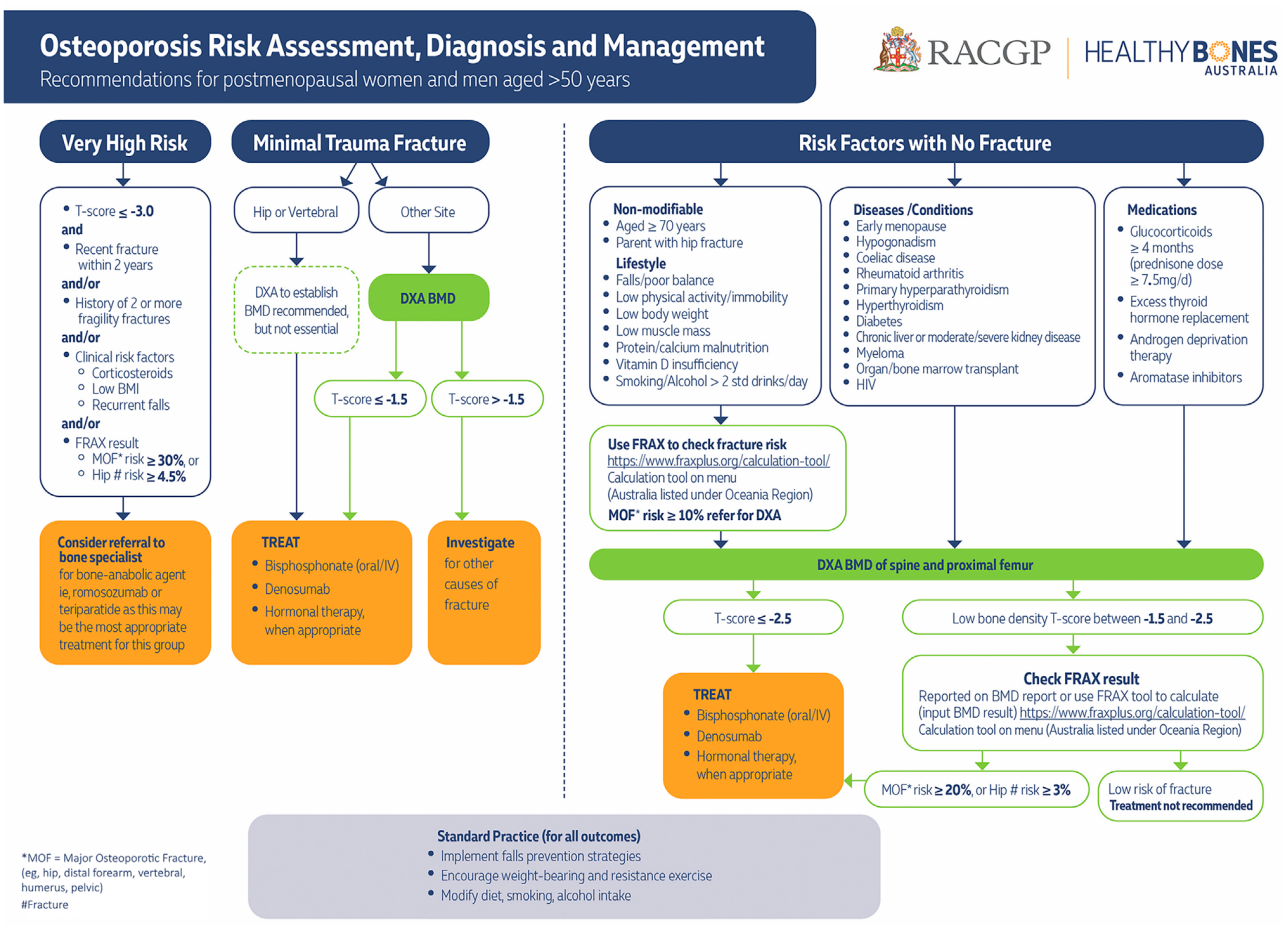

BMD = bone mineral density; BMI = body mass index; DXA = dual‐energy x‐ray absorptiometry; IV = intravenous.

## Clinical implications of updated recommendations

### Absolute risk calculation

Feedback received from general practitioners regarding the previous guideline was that they needed clear advice regarding which fracture absolute risk calculator to use in routine clinical practice. The Guideline Review Committee recognised the limitations of the FRAX tool (https://FRAXplus.org/calculation‐tool),^13^, ^14^ in particular, the use of binary (yes/no) responses for some inputs, the exclusion of falls as an input, and the lack of an open source calculation algorithm.^15^ However, many international guidelines suggest its use^16^, ^17^ because of validation in multiple populations, inclusion in many DXA reports, the regular algorithm refinement following user feedback,^18^ and the consideration of death as a competing hazard, meaning that fracture risk is reduced in people with low life expectancy (eg, older, frailer people). For these reasons FRAX was recommended, although clinical judgement remains essential for interpretation and communication of the ten‐year fracture risk output to patients. However, the simplicity of the Garvan Fracture Risk Calculator (only five input factors), which includes falls as one of the inputs, makes it very convenient, especially for patients who experience a fall.^19^

### Case finding

Since the previous guideline edition, there have been three large population‐based randomised controlled trials of screening in women for prevention of osteoporotic fractures: Screening in the Community to Reduce Fractures in Older Women (SCOOP) in the United Kingdom,^20^ Risk‐stratified Osteoporosis Strategy Evaluation (ROSE) in Denmark,^21^ and the SALT Osteoporosis Study (SOS) in The Netherlands.^22^ Although none showed a statistically significant reduction in the primary outcome of all fractures, there was a trend to a reduction. The planned secondary endpoint of a reduction in hip fractures showed a significant result in one trial and consistent non‐statistically significant improvements in the other two. This resulted in a significant result for hip fracture reduction in a meta‐analysis (total number of patients included, > 42 000).^23^ Although promising, optimal thresholds of absolute fracture risk and implementation strategies are inadequately defined for Australia and there are no data on screening in men. Therefore, the Guideline Review Committee concluded that there is insufficient evidence to support a population‐based screening program in Australia.

### A rational approach to assessment and BMD testing

Risk factors can be considered to better understand an individual's risk through the use of a fracture absolute risk calculator (Box 2). The screening trials used a two‐step process of initial FRAX risk assessment guiding the need for BMD measurement by DXA followed by repeat FRAX risk assessment, incorporating the BMD reading obtained at DXA to guide treatment recommendations.^20^, ^21^, ^22^ Use of a risk estimation tool, such as FRAX, also removes the need to set different minimum ages for initial risk enquiry for men and women, as sex and age are part of the risk estimation algorithm.

The absolute risk at which to recommend DXA and the threshold for commencement of pharmacotherapy is important, yet not consistently defined. The level of risk perceived as “high” will vary between individuals and will differ depending on regional regulatory body funding. Clinical trial results enable an estimate of absolute risk at which treatment is effective. In the Fracture Intervention Trial (FIT), where oral alendronate was effective at reducing fractures, almost all patients had a baseline ten‐year fracture risk greater than 10%.^24^ In the Fracture Reduction Evaluation of Denosumab in Osteoporosis Every Six Months (FREEDOM) trial of denosumab, which is a monoclonal antibody that inhibits receptor activator of nuclear factor‐κβ ligand [RANKL]), the median baseline ten‐year fracture risk was 15%.^25^ A trial of zoledronate–zoledronic acid in women with osteoporosis over 65 years old was effective, with a median baseline absolute risk of 12% for fracture at ten years.^26^

The thresholds used in the screening trials can also inform this choice. The ROSE study^21^ used a ten‐year fracture risk (FRAX) threshold of 15% to recommend DXA testing. The SCOOP^20^ trial used a range of age‐specific thresholds (3.4% at 50 years, rising to an 11.1% ten‐year risk of major osteoporotic fracture at 70 years), which might make implementation in the Australian primary care setting difficult without clinical decision support software for risk thresholds by age.

Given that case finding would be used for a population selected for their interest to engage in fracture prevention interventions, the impact can be expected to be better than demonstrated in population screening trials. A slightly lower threshold for recommending BMD has been adopted, as was done in the SIGN guidelines 2021, where a ten‐year risk of major osteoporotic fracture of more than 10% triggers a recommendation for BMD measurement, which is relatively pragmatic and inclusive.^27^ Patient preferences and value placed on a risk estimate should also guide further management.

A helpful guide to DXA testing, including appropriate Medicare Benefits Schedule item numbers, is provided in the guideline (appendix C)^10^ and is shown in the Supporting Information, figure 1.

### Imminent and very high fracture risk

The concept of imminent and very high fracture risk is evolving. The increased risk of refracture within the first 24 months following incident fracture means this is a crucial period in which to perform bone health assessment. Identifying patients with “very high” fracture risk is important with the increasing availability of osteoanabolic therapy, as these patients are a logical group in which to consider the use of these agents as initial therapy, subject to regulatory and funding limitations. The following features provide a broad guide as to which patients might be at “very high” fracture risk: (i) recent fracture and a ten‐year FRAX major osteoporotic fracture risk of 30% or over;^16^ (ii) a recent fracture (within 12 months); (iii) a T‐score below ‐3.0; (iv) multiple fractures while on therapy; (v) the use of drugs causing skeletal harm; and (vi) a ten‐year FRAX major osteoporotic fracture risk of 30% or above, or hip fracture risk of more than 4.5%.^17^ As outlined in the summary flowchart (Box 2), and available at https://www.racgp.org.au/getattachment/f31c6529‐96f0‐4840‐8f41‐c98bd5e4fad7/attachment.aspx?disposition=inline, such patients should be considered for referral to a bone specialist for consideration of early osteoanabolic therapy (romosozumab or teriparatide) followed by antiresorptive therapy.

### Calcium, vitamin D and protein supplementation

The use of calcium, vitamin D and protein supplementation is a complex area, with a vast amount of published literature discussed in the updated guideline.^10^ The absolute benefit of calcium and vitamin D supplementation for short term (less than five years) fracture prevention for non‐institutionalised individuals is relatively low and much less than with pharmacological treatments, such as bisphosphonates or denosumab.^28^, ^29^ The United States Preventive Services Task Force has recommended against routine calcium and vitamin D supplementation in non‐institutionalised older people.^30^ However, a comprehensive umbrella review concluded there was reasonable benefit for those who may be deficient, especially in institutionalised individuals or frail older people.^31^

The target calcium intake from dietary sources and supplements should be 1000 mg per day for adults, rising to 1300 mg per day for women aged over 50 years and men aged over 70 years.^28^, ^32^, ^33^ Vitamin D can be obtained from sunlight exposure or, if sun exposure is limited, supplements should ensure a serum 25‐hydroxyvitamin D (25(OH)D) concentration of more than 50 nmol/L.^28^ If oral vitamin D supplements are required,^28^ a dose of 800–1000 IU per day is usually sufficient.

Calcium supplements modestly increase the risk of renal calculi and can cause abdominal bloating and constipation.^34^ Although an increased risk of myocardial infarction with calcium supplements has been reported,^35^ not all studies support this conclusion.^36^, ^37^ However, obtaining an adequate calcium intake by dietary means is preferable.

Findings from the large Vitamin D and Omega‐3 Trial (VITAL) study were recently published and cast doubt on the role of vitamin D3 supplementation in fracture risk reduction.^38^ However, this study was done in generally healthy mid‐life and older adults who were not selected for vitamin D deficiency, low bone mass, or osteoporosis.

Based on a large body of evidence over many years, calcium and vitamin D supplements are more likely to be effective in reducing fracture risk when given in combination to individuals who are deficient (serum 25(OH)D < 50 nmol/L). It is important to note that most pharmacological intervention studies were done in calcium‐ and vitamin D‐replete individuals. In healthy non‐institutionalised individuals, the relative reduction in fracture risk with calcium and/or vitamin D supplementation alone is small and, thus, these should not be considered for routine use in healthy people or as first line treatment for people with osteoporosis.

An authoritative position statement, *Balancing the harms and benefits of sun exposure*,^39^ by the Australian Skin and Skin Cancer Research Centre and endorsed by a wide range of stakeholders, was released as this guideline was being updated. This provides practical advice about the duration of sunlight exposure required for adequate skin production of vitamin D (vitamin D‐effective dose of sunlight) in people with diverse skin tones residing in various geographic locations around Australia. As the risks and benefits of sun exposure are mainly determined by skin type and risk of skin cancer, the recommendations are stratified as follows:

- **Individuals at high risk of skin cancer (eg, those with very pale skin)**. In this group, time outdoors with an ultraviolet (UV) index (a measure of UV radiation ranging from 0 [low] to 11 [extremely high]) ≥ 3 should be avoided, and if outdoors at those times, full sun protection measures should be implemented — “Slip on covering clothing. Slop on sunscreen with a sun protection factor [SPF] ≥ 30. Slap on a hat. Seek shade. Slide on sunglasses”.
- **Individuals at low risk of skin cancer (eg, those with dark skin)**. These people should be advised to spend sufficient time outdoors with enough skin exposed when the UV index is ≥ 3.
- **Individuals at intermediate risk of skin cancer (eg, those with olive or pale brown skin and no other risk factors)**. These people should be advised to spend enough time outdoors with sufficient skin exposed for a vitamin D‐effective dose of sunlight. Full sun protection measures (see above) should be used if spending more time than that required to obtain a vitamin D‐effective dose.^39^

Readers requiring exposure times for a vitamin D‐effective dose of sunlight should refer to the detailed seasonal charts in the position statement for their specific geographic area of practice.^39^

The importance of protein supplementation was highlighted by an influential Melbourne study that assessed the effectiveness of a nutritional intervention in institutionalised older adults by improving calcium and protein intake (< 1 g/kg body weight protein per day) using dairy foods. This study showed an 11% reduction in falls risk, a 48% reduction in hip fractures, and a 30% reduction in all fractures in the intervention group.^40^

In summary, supplementation with calcium (target intake > 1300 mg per day), vitamin D (target serum 25(OH)D > 50 nmol/L) and protein (1–1.2 g/kg body weight per day) should be targeted to people who need it most, namely frail, institutionalised individuals, especially those receiving bone‐protective therapy.

### Osteoanabolic therapy

The current Pharmaceutical Benefit Scheme (PBS)‐subsidised indications for bone protective therapy are outlined in the Supporting Information, table 2.^41^ Therapeutic options for poor bone health have changed since the previous guideline, with removal of strontium ranelate due to associated excess cardiovascular mortality,^42^ and addition of the sclerostin inhibitor, romosozumab, which increases bone formation and reduces bone resorption. This novel dual mechanism of action leads to a marked increase in BMD, greater than that seen with oral alendronate (bisphosphonate) or teriparatide (recombinant human parathyroid hormone (1‐34) [rhPTH (1‐34)]).^43^, ^44^ Concern about a small increase in cardiovascular adverse events compared with alendronate in one randomised controlled trial^45^ with several local notifications of adverse events prompted the Therapeutic Goods Administration to issue an alert that romosozumab should be avoided in individuals with previous myocardial infarction or stroke.^46^ As always, its use requires discussion with the individual patient regarding risk and benefit, especially as the same ageing population at risk of poor bone health is also at increased risk of adverse cardiovascular events.

The other osteoanabolic agent available via the PBS subsidy is rhPTH (1‐34), teriparatide (Supporting Information, table 2). Withdrawal of the originator compound, Forteo (Eli Lilly) has left the biosimilars Terrosa (Gedeon Richter) and Teriparatide Lupin (Generic Health) as the only teriparatide formulations in Australia. Prescription (total duration of therapy, 18 months) can be continued by a general practitioner following initiation by a bone specialist.

Wider access to osteoanabolic therapies (romosozumab and rhPTH (1‐34)) has prompted identification of patients at “very high” fracture risk (see above and Box 2), as these should be considered for early osteoanabolic therapy followed by an antiresorptive agent, subject to regulatory and funding restrictions.

As of 1 November 2024, romosozumab received a first line PBS listing for patients at very high fracture risk — the detailed statement of the PBS indication is presented in the Supporting Information, table 2.^41^ This will allow initiation of potent bone anabolic therapy in treatment‐naïve patients to be sequentially followed by antiresorptive therapy (eg, a bisphosphonate or denosumab) to achieve and maintain the greatest possible gain in BMD.^45^

### Transition of bone protective agents

Although the advent of denosumab has been a major advance in the treatment of osteoporosis, its discontinuation, or even delaying the injection by more than four months can be associated with rebound bone resorption and vertebral fractures.^47^, ^48^ Even though definitive measures to prevent this remain unclear, denosumab should either be continued long term or its cessation followed by an antiresorptive medication; for example, 12 months of an oral bisphosphonate or one or more infusions of zoledronate–zoledronic acid (a potent intravenous bisphosphonate).^49^, ^50^ Most general practices have a robust recall system to ensure denosumab administration occurs at the specified six‐monthly intervals to minimise the risk of rebound vertebral fractures.

### Implementation

The complete guideline can be accessed at no cost from the following professional society websites:

- RACGP (https://www.racgp.org.au/clinical‐resources/clinical‐guidelines/key‐racgp‐guidelines/view‐all‐racgp‐guidelines/osteoporosis/executive‐summary);
- Australian Rheumatology Association (https://rheumatology.org.au/For‐Healthcare‐Professionals/Clinical‐Resources/Other); and
- Australian and New Zealand Bone and Mineral Society (https://www.anzbms.org.au/policies.asp).

It can also be accessed from the Healthy Bones Australia website (https://healthybonesaustralia.org.au/health‐care‐professionals/gp‐resources/).

## Conclusion

Poor bone health (osteopenia and osteoporosis) is highly treatable with appropriate widely available lifestyle and dietary measures and pharmacological agents. As general practice is the only extensive workforce capable of long term care of patients with osteoporosis, supporting general practitioners to manage osteoporosis is critical. The updated guideline is designed to be an evidence‐based pragmatic tool to assist general practitioners in the day‐to‐day care of such patients in partnership with bone specialists.

## Open access

Open access publishing facilitated by The University of Sydney, as part of the Wiley ‐ The University of Sydney agreement via the Council of Australian University Librarians.

## Competing interests

Peter Wong is a member of the Clinical Advisory Group, NSW Health, and Osteoporosis Refracture Working Group (no remuneration attached). He is also the site investigator for a phase 4 post‐marketing Amgen clinical trial (remuneration only to the institution). Weiwen Chen was a speaker for the educational seminar for Arrotex as part of GP Health Update Day (31 Aug 2024) and was a member of the Sandoz Advisory Board on one occasion (11 May 2024). Christian Girgis was a member of the Advisory Board for Sandoz on one occasion (11 May 2024) and was a speaker for Gedeon Richter (12 Sept 2023). All other members of the Guideline Review Committee declared no relevant competing interests.

## Provenance

Not commissioned; externally peer reviewed.

## Supporting information


Supplementary figure and tables
