# Supplementary material for: 2024 Royal Australian College of General Practitioners and Healthy Bones Australia guideline for osteoporosis management and fracture prevention in postmenopausal women and men over 50 years of age
Source: Med J Aust. 2025 Mar 25;222(9):472–80. doi: 10.5694/mja2.52637 (PMC12088310; doi:10.5694/mja2.52637)
Supplement: Supplementary file 1 — Supplementary figure and tables [file MJA2-222-472-s001.pdf]

Supporting Information

Supplementary material

This appendix was part of the submitted manuscript and has been peer reviewed.
It is posted as supplied by the authors.

Appendix to: Wong P, Chen W, Ewald D, et al. 2024 Royal Australian College of General Practitioners and Healthy Bones Australia guideline for osteoporosis management and fracture prevention in postmenopausal women and men over 50 years of age. *Med J Aust* 2025; doi: 10.5694/mja2.52637.

Table 1. Subject Matter Advisers

Name	Qualifications	Positions
Professor Belinda Beck	BMHS (Ed), MS, PhD, FACSM	Director, The Bone Clinic Brisbane; Professor, Exercise Science, Griffith University, Queensland
Professor Peter Ebeling AO	MBBS, MD, FRACP, FAHMS	Endocrinologist and Head, Monash University Department of Medicine, Monash Health, Victoria; Board Chair, Healthy Bones Australia; Board Member International Osteoporosis Foundation; Past President, Australian and New Zealand Bone and Mineral Society and Endocrine Society of Australia; Past President, American Society of Bone and Mineral Research
Professor Jacqueline Close AM	MD, FRCP, FRACP	Consultant Geriatrician, Prince of Wales Hospital, Sydney and Clinical Director of Falls, Balance and Injury Research Centre, Neuroscience Research Australia; Co-Chair, Australian and New Zealand Hip Fracture Registry, Chair of the Australian Commission on Safety and Quality in Health Care Clinical Care Standards Working Group for Hip Fracture
Professor Robin Daly	PhD, FSMA	Chair of Exercise and Ageing, Institute for Physical Activity and Nutrition, School of Exercise and Nutrition Sciences, Faculty of Health, Deakin University, Victoria
Professor Mathis Grossmann	MD, PhD, FRACP	Professor, Department of Medicine, the University of Melbourne; Head of Andrology, Austin Health, Victoria
Clinical Professor Charles Inderjeeth	MBChB, MPH, FRACP	Clinical Professor, University of Western Australia; Clinical Dean, Curtin University; Consultant Physician (geriatrician and rheumatologist); National Health and Medical Research Council Fellow and Clinical Epidemiologist; Director of Clinical Training, Research and Programs, Osborne Park Hospital, Western Australia
Professor Stephen Lord	PhD, DSc	Senior Principal Research Fellow, NeuRA; Professor, School of Community Medicine and Public Health, UNSW Sydney, New South Wales
Professor Rebecca Mason AM	MBBS (Hons), PhD	Professor of Endocrine Physiology, School of Life and Environmental Sciences, University of Sydney, New South Wales
Associate Professor Nicholas Pocock	MBBS, MD, FRACP	Visiting Scientist, Garvan Institute of Medical Research; Associate Professor of Medicine (Conjoint), UNSW Sydney; Senior Staff Specialist in Nuclear Medicine, St Vincent's Hospital Sydney, New South Wales
Professor Richard Prince	BSc, MB ChB (Birm), MD, FRACP, MRCP (UK)	Senior Honorary Research Fellow, Medical School, University of Western Australia, Western Australia
Dr Ayse Zengin	PhD	Senior Research Fellow, Monash University Department of Medicine, Monash Health, Victoria

Table 2. Current Pharmaceutical Benefits Scheme-subsidised indications for osteoporosis therapy[1]

Medications	Pharmaceutial Benefits Scheme indications
**Antiresorptives**	
Risedronate or alendronate	▪ ≥ 3 months of prednisone ≥ 7.5 mg/day + T-score ≤ –1.5<br>▪ Patients aged ≥ 70 years + T-score ≤ –2.5<br>▪ MTF
Zoledronic acid	▪ ≥ 3 months of prednisone ≥ 7.5 mg/d + T-score ≤ –1.5<br>▪ Patients aged ≥ 70 years + T-score ≤ –3.0<br>▪ MTF
Denosumab	▪ Patients aged ≥ 70 years + T-score ≤ –2.5<br>▪ MTF
**Bone anabolic agents**	
Teriparatide (18-month duration)	▪ T-score ≤ –3.0 + ≥ 2 MTFs +1 symptomatic new fracture after ≥ 12 months of continuous antiresorptive therapy
Romosozumab (12-month duration)	
First line therapy	▪ T-score ≤ –2.5 + symptomatic MTF + either ≥ 1 hip or symptomatic vertebral fracture in previous 24 months OR<br>▪ ≥ 2 fractures, including 1 symptomatic new fracture in previous 24 months
Second line therapy	▪ T-score ≤ –3.0 + ≥ 2 MTFs +1 symptomatic new fracture after ≥ 12 months of continuous antiresorptive therapy

MTF = minimal trauma fracture.

1. Australian Government Department of Health and Ageing. Pharmaceutical Benefits Scheme. http://www.pbs.gov.au/ (viewed Nov 2024).

Figure 1. Bone density testing by dual energy x-ray absorptiometry (DXA) with Medicare Benefits Schedule (MBS) item numbers

HEALTHY BONES AUSTRALIA

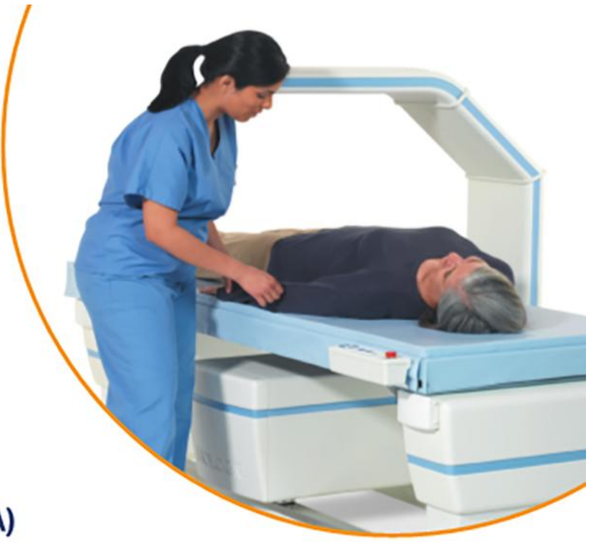

Bone density testing in general practice

A guide to Dual Energy X-ray Absorptiometry (DXA)

Scanning of the axial skeleton by dual energy X-ray absorptiometry (DXA) is the gold standard in Australia for the measurement of bone mineral density (BMD). DXA is a diagnostic tool for osteoporosis or osteopenia, enabling doctors to determine the extent of bone loss for clinical decision making. This guide outlines who to refer for DXA and the basics of how to interpret a bone densitometry report. Note the terminology of osteopenia and osteoporosis based on BMD alone in intended for individuals over 50 years of age and menopausal women.

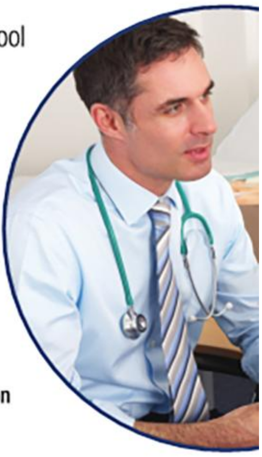

Poor bone health is common in Australia

An estimated 4.7 million Australians over the age of 50 years have osteoporosis or osteopenia, with over 183,000 associated fractures (2022). Early diagnosis and improved management can reduce the current annual cost of $3.84 billion, with fracture costs accounting for up to 67% of overall costs.

In general practice, early detection can prevent a first fracture. For patients who have already fractured, investigation and initiation of osteoporosis medication is crucial to reduce the very high risk of subsequent fractures.

Who to send for a DXA scan

Patients over 50 with risk factors	MBS item
Family history – parent with hip fracture	No rebate
Early menopause	12312
Hypogonadism	12312
Anticipated glucocorticoids ≥4 months ≥7.5mg/day	12312
Coeliac disease/malabsorption disorders	12315
Rheumatoid arthritis	12315
Primary hyperparathyroidism	12315
Hyperthyroidism	12315
Chronic kidney or liver disease	12315
Androgen deprivation therapy	12312
Recurrent falls	No rebate
Breast cancer on aromatase inhibitors	No rebate
Treatment with antiepileptic medications	No rebate
Low body weight	No rebate
HIV and its treatment	No rebate
Major depression/ SSRI treatment	No rebate
Type 1 and type 2 diabetes mellitus	No rebate
Multiple myeloma/monoclonal gammopathy	No rebate
Organ or bone marrow transplant (item 12312 applies if treated with glucocorticoids or if kidney disease present)	No rebate

Patients with a minimal trauma fracture	MBS item
DXA is recommended to establish a baseline BMD for treatment	12306
**Suspected vertebral fracture**	**MBS item**
Refer for spinal X-ray when:<br>– Height loss of 3cm or more<br>– Thoracic kyphosis<br>– New onset back pain suggestive of fracture<br>**If fracture confirmed, therapy indicated, refer for DXA**	12306
Vertebral fracture assessment (VFA), also known as Lateral vertebral assessment (LVA) is offered with some DXA scans. VFA may be a useful screen for fractures in people with height loss. MBS rebate not available for VFA.	
**Patients with osteoporosis**	**MBS item**
T-score equal to or less than -2.5 eligible for one scan every two years	12306
**Patients over 70 years of age**	**MBS item**
For men and women over 70 years, MBS rebate applies (regardless of other risk factors)	12320
Patients with a normal result or mild osteopenia (measured by a T-score down to -1.5) eligible for one scan every 5 years	12320
Patients with moderate to marked osteopenia (as measured by a T-score less than -1.5 and above -2.5) will be eligible for one scan every two years	12322
